# Context and clinical reasoning: Understanding the medical student perspective

**DOI:** 10.1007/s40037-018-0417-x

**Published:** 2018-04-27

**Authors:** Elexis McBee, Temple Ratcliffe, Lambert Schuwirth, Daniel O’Neill, Holly Meyer, Shelby J. Madden, Steven J. Durning

**Affiliations:** 1 Department of Medicine, F. Edward Hébert School Of Medicine, Uniformed Services University, at Naval Medical Centre San Diego, San Diego, CA USA; 2 Department of Medicine, University of Texas Health Science Centre at San Antonio, San Antonio, TX USA; 3 School of Medicine, Flinders University, Adelaide, South Australia Australia; 4 Department of Medicine, F. Edward Hébert School Of Medicine, Uniformed Services University, Bethesda, MD USA

**Keywords:** Clinical reasoning, Medical education, Situated cognition, Qualitative methods

## Abstract

**Introduction:**

Studies have shown that a physician’s clinical reasoning performance can be influenced by contextual factors. We explored how the clinical reasoning performance of medical students was impacted by contextual factors in order to expand upon previous findings in resident and board certified physicians. Using situated cognition as the theoretical framework, our aim was to evaluate the verbalized clinical reasoning processes of medical students in order to describe what impact the presence of contextual factors has on their reasoning performance.

**Methods:**

Seventeen medical student participants viewed three video recordings of clinical encounters portraying straightforward diagnostic cases in internal medicine with explicit contextual factors inserted. Participants completed a computerized post-encounter form as well as a think-aloud protocol. Three authors analyzed verbatim transcripts from the think-aloud protocols using a constant comparative approach. After iterative coding, utterances were analyzed and grouped into categories and themes.

**Results:**

Six categories and ten associated themes emerged, which demonstrated overlap with findings from previous studies in resident and attending physicians. Four overlapping categories included emotional disturbances, behavioural inferences about the patient, doctor-patient relationship, and difficulty with closure. Two new categories emerged to include anchoring and misinterpretation of data.

**Discussion:**

The presence of contextual factors appeared to impact clinical reasoning performance in medical students. The data suggest that a contextual factor can be innate to the clinical scenario, consistent with situated cognition theory. These findings build upon our understanding of clinical reasoning performance from both a theoretical and practical perspective.

## What this paper adds

The traditional view is that contextual factors are extrinsic to clinical reasoning performance (e. g. they are noise). Recent studies have demonstrated that contextual factors influence clinical reasoning performance in resident and board certified physicians. Student clinical reasoning performance has not been examined and doing so could lend insight into findings of expert performance development and context specificity. The data suggest that contextual factors can be innate to the clinical scenario, consistent with situated cognition. This raises important questions about how contextual factors are processed by students and physicians.

## Introduction

Clinical reasoning involves establishing a diagnosis and developing a therapeutic plan that fits the unique circumstances and needs of the patient [1, 2]. Clinical reasoning requires an interplay of effortful processing of a patient’s symptoms and various physical, laboratory and/or radiographic findings (analytic reasoning) and thinking that is typically subconscious and rapid (nonanalytic reasoning), to ensure the optimal outcome is reached for each patient. To properly study clinical reasoning in a clinical encounter, the potential impact of the unique context of each encounter, or contextual factors, should be taken into account. This idea is supported by the finding of context specificity, meaning that a physician can see two patients with the same symptoms, findings, and seemingly the same diagnosis, and yet he/she arrives at two different diagnoses [3–5].

**Fig. 1:**
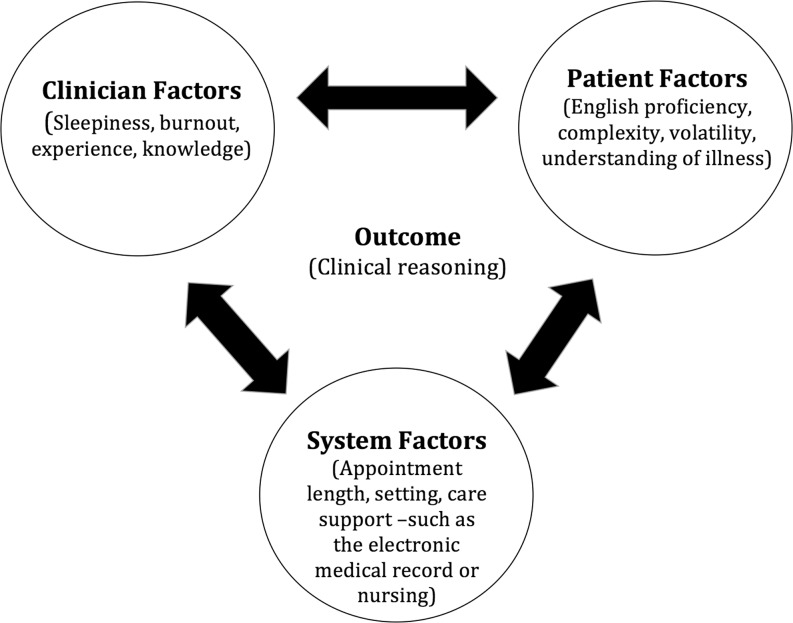
Situated cognition and clinical reasoning—a theoretical framework. The clinical outcome is dependent on the interplay between the patient, the environment, and the clinician. The circles show the relationship between all individual factors, while the centre portion represents the clinical outcome (clinical reasoning) being affected by all three. Situated cognition suggests that the individual and their environment can influence an outcome. In the case of a clinical environment, the outcome would involve the patient. (Note: the diagram simplifies the numerous interactions that can occur between the various factors within and between the various circles)

Situated cognition theory (Fig. 1) provides a lens for exploring the situation-dependent nature of clinical reasoning, accounting for the multiple interactions that occur between the physician, the patient, and the environment during a clinical encounter [6–8]. All of these dimensions of context are thought to be intrinsically linked and emerge to inform the outcome, in this case, clinical reasoning. Thus, situated cognition theory contends that clinical reasoning is a non-linear process that is a by-product of multiple interactions that occur during an encounter. This theory contrasts with other theories, such as dual processing, which emphasizes the individual and minimizes the role of social, physical or environmental interactions. Previous studies have demonstrated how contextual factors, such as low English proficiency and diagnostic suggestion, affected residents’ and attending physicians’ clinical reasoning performance utilizing the same study design [2, 6]. For example, resident physicians demonstrated universal acknowledgement of the presence of contextual factors during clinical encounters and ultimately experienced difficulty with diagnostic uncertainty [6]. However, medical student clinical reasoning performance has not been examined from a situated cognition perspective and doing so could lend insight into the finding of expert performance development as well as context specificity. Studying medical students may also allow educators to better inform their clinical reasoning curricula and assessment methods.

In this study, using the lens of situated cognition, we investigated the thought processes of medical students exposed to selected contextual factors in order to understand how such contextual factors impact their clinical reasoning performance. We then compared these findings with previous findings from both board certified internists and resident physicians who viewed the same video recordings to also help inform the expertise continuum. It was predicted that medical students’ clinical reasoning performance would be impacted by a wider variety of factors than either of these groups. We also anticipated that the use of our social cognitive approach, which embraces complexity and the common ‘stimulus’ of learners across the continuum, would yield new understandings to help inform clinical reasoning theory and educational practice.

## Methods

### Participants

Medical students from the Uniformed Services University were invited to take part in this study from 2015 to 2016. All third and fourth year students (230 in total) were invited by email by a research assistant to participate. There were no exclusion criteria. As part of the standard curriculum at our institution, in the pre-clinical years all students have instruction on clinical reasoning in both lecture and small group discussion formats in an Introduction to Clinical Reasoning course. In addition, they also receive additional practice in a simulation centre in an Integrated Clinical Skills course. The study was approved by the institutional review board at the Uniformed Services University of the Health Sciences, protocol number R083VC-03.

### Design

The design of this study was modelled after our previous research [2, 6]. Participants viewed a series of three video recordings (Tab. 1) that displayed a clinical encounter with one of the three predetermined diagnoses: Human immunodeficiency virus (HIV), colorectal cancer, or symptomatic type 2 diabetes mellitus. Each video case was designed to represent a straightforward disease presentation of the desired diagnosis. These videos were reviewed by an independent group of physicians who were not involved in the study for verification of authentic portrayal of said diseases. As the material covered in the cases is part of our core curriculum and the depictions of the videos are classic/straightforward presentations of these diseases, post clerkship medical students in the curriculum at our university would be expected to recognize the correct diagnosis for each video. Medical students viewed these videos but did not have access to any other clinical information (e. g. written medical records) about the patients portrayed. Specific patient contextual factors were: (1) a patient with low English proficiency, diagnosis of HIV, (2) a patient with emotional volatility (challenging of the physician’s credentials), diagnosis of colorectal cancer and (3) a combination of both of these contextual factors, diagnosis type 2 diabetes mellitus (Tab. 1).

**Table 1:** Video recorded clinical encounters with modified contextual factors used to evaluate clinical reasoning in medical students

Clinical encounter	Diagnosis	Contextual factor/s modified
Case One	HIV	English as a second language
Case Two	Colorectal cancer	Emotional volatility (Challenging of the physician’s credentials)
Case Three	Diabetes mellitus	English as a second language + emotional volatility

Participants watched each video and then completed a post-encounter form [9, 10] that was validated for capturing clinical reasoning processes in a previous study which queried about additional history and physical examination information, differential diagnosis, leading diagnosis, and management plan. Immediately following completion of the post-encounter form students re-watched the video and participated in a think-aloud protocol [11]. During the think aloud, students stated whatever came to mind, and if no utterances were vocalized after a period of ten seconds, a research assistant would ask the participants to ‘think aloud.’ The think-aloud sessions were audio recorded and transcribed verbatim into written transcripts.

### Data analysis

The qualitative data from the think-aloud transcripts were analyzed using a constant comparative approach [12]. An aspect of grounded theory, open coding searched for emerging themes. Although the researchers had been informed by the situated cognition framework, we attempted to remain as open minded as possible during coding. Four investigators (EM, TR, SJD, SHM) conducted iterative coding of utterances (single words, phrases, or sentence(s) corresponding to a theme) and then grouped these utterances into categories and themes (see results below). Following initial coding, four investigators (EM, TR, SJD, SHM) met to discuss themes and resolve differences. Several themes emerged and the process continued until saturation was reached. Investigators met for a final time to review the categories and themes and to resolve all differences in coding until 100% agreement was met. The researchers then reviewed these findings and compared and contrasted them with prior findings with practising physicians and residents. Three coders in this study (EM, TR, SJD) participated in the coding of resident physicians and one author (SJD) also coded the comments of the attending physicians.

Descriptive statistics for demographic data was also reported using SPSS 22.0.

## Results

A total of 17 third or fourth year medical students participated in the study. The mean age of the participants was 29 (standard deviation = 5.25) and 78% of the participants were male. Saturation was reached after coding of half of the transcripts.

As in previous studies [2, 6], all students verbalized the presence of the contextual factors in every case. Students made statements that ranged from (a) an acknowledgment to (b) specifically addressing the contextual factor:

(a) ‘She’s very sassy in the beginning.’ (Case 2, colorectal cancer, emotional volatility)

(b) ‘What is the best way for a doctor to talk to someone who doesn’t speak English?’ (Case 3, diabetes mellitus, emotional volatility and low English proficiency).

All emerging concepts were organized into six categories with 10 corresponding themes (Tab. 2). The six categories were: (1) emotional reactions by the student, (2) behavioural inferences about the patient, (3) doctor-patient relationship, (4) difficulty with closure (3 themes), (5) anchoring (two themes), and (6) misinterpretation of data (two themes). The categories of anchoring and misinterpretation of data, with the corresponding themes, were findings not seen in the previous resident and attending physician studies [2, 6]. A description of the categories and themes is listed below.

**Table 2:** Categories and themes identified from think-aloud transcripts of medical students (Multiple constructs emerged from the data that appeared to be the consequence of contextual factors during the clinical encounter. These were grouped into six categories with associated themes.)

Categories	Themes	Example quote
Emotional reactions by the student	Emotional reactions to contextual factors	‘Her lack of eye contact in this interview is disturbing to me*.*’
Behavioural inferences about the patient	Behavioural inferences made in response to contextual factors	‘She sounds lonely.’
Doctor-patient relationship	Identifying that the contextual factor may hinder the doctor patient relationship	‘She is still frustrated so he (the doctor) probably needs to address that.’
Difficulty with closure	Need for additional history and physical exam information	‘How much exercise does she do? Tired? What does that mean?’
	Inability to utilize presented information	‘What is that spot on her leg?’
	Presence of uncertainty of clinical reasoning	‘So my diagnosis would probably be infectious aetiology.’
Anchoring	Displayed tendency to anchor on first diagnosis	‘So four years is a long time ago (prior head injury), but either way diabetes insipidus is what I’m thinking for my lead diagnosis.’
	Limited differential diagnosis	–
Misinterpretation of data	Factual errors	‘Lack of insulin means she probably can’t digest her food. Keeps you hungry.’
	Limitations in knowledge	‘I don’t know anything about feminine care so refer her to OB/GYN.’

### Emotional reactions by the student to contextual factors

During the think-aloud process, participants verbalized a variety of emotional reactions in response to the contextual factors. Their verbalized emotions appeared to reveal internal tension and often implied a general judgment about the patient. For example:

‘Her lack of eye contact in this interview is disturbing to me.’ (Case 2, colorectal cancer, emotional volatility)

‘This lady is annoying.’ (Case 2, colorectal cancer, emotional volatility)

### Behavioural inferences about the patient made in response to contextual factors

Medical students demonstrated inference about the patient’s lifestyle behaviours, emotional state, and the patient’s understanding of their symptoms based in response to the contextual factor. While not every participant demonstrated making a behavioural inference about a patient, when present, it was most often of a negative connotation.

‘She sounds lonely.’ (Case 1, HIV, low proficiency in English)

‘Need to address her issues with trust. She sounds anxious.’ (Case 2, colorectal cancer, emotional volatility)

### Impact on the doctor-patient relationship

Participants recognized the presence of a contextual factor and often commented on how they thought the contextual factor contributed to a poor relationship between the doctor and patient. Subsequently, participants then commented on how the physician could have acted to improve the relationship.

‘She is still frustrated so he (the doctor) probably needs to address that.’ (Case 2, colorectal cancer, emotional volatility)

‘She overacted. He (the doctor) should have explained himself.’ (Case 1, HIV, low proficiency in English)

### Difficulty with closure

Difficulty with closure manifested as the presence of diagnostic uncertainty and the inability to commit to a final diagnosis. Three associated themes emerged with difficulty with closure to include (1) the need for additional history and physical examination information, (2) the inability to utilize presented information, and (3) presence of uncertainty in clinical reasoning.

The first theme, the need for additional history and physical examination, manifested as the participants expressing they desired more detail from the patients while watching the videos. Further information appeared to be needed (or at least desired) in order for students to commit to a diagnosis.

‘How much exercise does she do? Tired? What does that mean?’ (Case 2, colorectal cancer, emotional volatility)

‘Need to ask other questions, like loss of sensation in feet.’ (Case 3, diabetes mellitus, low proficiency in English and emotional volatility)

The second emerging theme, the inability to utilize presented information, related to the difficulty participating students had with closure due to limits in their comprehension. This appeared to be due to a relative lack of knowledge and clinical experience.

‘What is that spot on her leg?’ (Case 1, HIV, low proficiency in English)

‘Why is she here right now?’ (Case 2, colorectal cancer, emotional volatility)

‘I am feeling a little bit confused.’ (Case 3, diabetes mellitus, low proficiency in English and emotional volatility)

The third theme was the explicit presence of uncertainty in clinical reasoning. The participating students expressed doubt regarding their lead diagnosis or were unable to come to a definitive diagnosis. This was greatest in Case 1 and least in Case 3.

‘So my diagnosis would probably be infectious aetiology.’ (Case 1, HIV, low proficiency in English)

‘So it’s definitely not the flu, could be the flu? Probably not the flu.’ (Case 1, HIV, low proficiency in English)

### Anchoring

Anchoring was one of two categories not identified in prior work with resident and expert physicians [2, 6]. At times, students displayed a clear tendency to anchor on their first diagnosis seeming to underestimate the value of new data that were presented due to preconceived assumptions. For example, in Case 1 (HIV, low proficiency in English) some students discounted the presence of a new skin lesion (Kaposi sarcoma) in favour of a diagnosis of strep throat when consideration of the new information presented should have increased suspicion for HIV in this clinical context and altered clinical reasoning. In addition, the differential diagnoses that were generated remained limited. Across cases the mean number of differential diagnoses generated for Case 1 was three and for Cases 2 and 3 was four. For example:

‘So four years is a long time ago (prior head injury), but either way diabetes insipidus is what I’m thinking for my lead diagnosis.’ (Case 3, diabetes mellitus, low proficiency in English and emotional volatility)

### Misinterpretation of data

The misinterpretation of data was the second category not seen in the previous clinical reasoning studies with residents and board certified physicians. Two corresponding themes emerged: (1) factual errors and (2) limitations in knowledge. In the first theme, factual errors occurred when students fixated on clinical information that they misinterpreted.

‘She has got a bruise on the side of her leg.’ (Kaposi sarcoma in Case 1, HIV, low proficiency in English)

‘Lack of insulin means she probably can’t digest her food. Keeps you hungry.’ (Case 3, Diabetes mellitus, emotional volatility and low proficiency in English)

Limitations in knowledge appeared to be related to the student’s paucity of clinical experience. Participants would often defer care to a more knowledgeable professional or make assumptions based on their limited knowledge and experience. For example, in Case 3 (diabetes mellitus, emotional volatility and low proficiency in English), the patient tells the physician that she thinks she has a yeast infection. Instead of recognizing this as a possible complication of untreated diabetes mellitus that warrants treatment and will likely recur unless the diabetes mellitus is treated, the student states:

‘I don’t know anything about feminine care so refer her to OB/GYN.’

‘I don’t know if diabetes insipidus can show up four years after a head injury.’ (Case 3, diabetes mellitus, emotional volatility and low proficiency in English)

## Discussion

In this study, using the lens of situated cognition, we investigated the thought processes of medical students exposed to select contextual factors in order to explore how contextual factors impact their clinical reasoning performance. Using the identical protocol used in residents and board certified physicians, we anticipated that medical students’ clinical reasoning performance would be impacted by a wider variety of factors compared with these prior groups. This study adds to previous literature exploring how contextual factors impact clinical reasoning by examining themes that emerged across the range of physician experience. Although we found some similar themes across participant groups, the additional themes that emerged in medical students suggest that individual physician contextual factors (such as the robustness of illness script libraries and clinical experience) impact clinical reasoning in this participant population.

Medical student responses to contextual factors did overlap with both resident and board certified physicians. Consistent with prior groups [2, 6], medical students universally acknowledged the presence of the contextual factors and like resident physicians, identified their presence as a hindrance to the doctor-patient relationship. After acknowledgement, or with some explicitly addressing the contextual factor, students often verbalized an emotional reaction to the patient or made a behavioural inference. Subsequently, medical students often demonstrated diagnostic uncertainty and/or repeatedly expressed the need for additional history or physical exam information in order to establish a diagnosis despite these being straightforward cases that provided adequate information to make a sound diagnosis. Taken together, these findings provide further insight into how the presence of contextual factors can interfere with students’ clinical reasoning processes.

The exhibited emotional reactions in response to a contextual factor usually manifest as a judgment about the patient and, at times, led to behavioural inferences. Speaking in general terms, if a physician demonstrates an emotional reaction or makes a behavioural inference about a patient, this is not universally negative. If the emotional reaction or behavioural inference contributes to correct clinical decision-making, a negative inference may be justifiable and could even be key to establishing the diagnosis. For example, if a physician infers that a patient ‘sounds lonely’ (Case 2), and it is actually verified that the patient is in fact lonely, this may potentially contribute to a correct diagnosis of depression. However, if the emotional reaction or inference is not confirmed, incorrect, or tangential to the patient’s reason for presenting to the physician, this has the potential to impede clinical reasoning processes. In this study and consistent with prior work [2, 6], the tendency was for the development of a negative emotional reaction or behavioural inference in the setting of our contextual factors, which was neither verified by the portrayed physician nor the participant. As such, the presence of these contextual factors appeared to play a distracting role in the interpretation of the clinical interaction and could lead to changes in clinical reasoning performance through increases in cognitive load [13].

Also, similar to resident physicians, medical students had difficulty achieving diagnostic closure [6]. The minimum required task was to arrive at a lead diagnosis during the think-aloud protocol. Medical students demonstrated significant uncertainty and difficulty arriving at a final diagnosis, which was greatest for Case 1 and least in Case 3. In addition, medical students repeatedly expressed the need to acquire additional history and physical exam data. Script theory contends that physicians use prior gained, relevant knowledge during clinical encounters to generate hypotheses and actionable plans [14]. Together, this suggests that in the presence of contextual factors, physicians require the opportunity to ask their own questions in order to confirm the diagnosis according to their own individual illness scripts. The medical students, who have less robust illness scripts, have less knowledge and thus limitations in their strategies to deal with the clinical information presented in the case scenarios in this study impairing their ability to reach diagnostic closure. Diagnostic uncertainty was greater for case 1 (HIV) than for Case 3 (type 2 diabetes mellitus) despite the presence of two contextual factors in the latter case. The prevalence of type 2 diabetes mellitus is much higher in the United States than HIV and students are exposed to more cases of diabetes mellitus than HIV in the pre-clerkship curriculum. It may be that students demonstrated less uncertainty in Case 3, despite the presence of two contextual factors, as a result of greater disease exposure leading to a more robust illness script that was able to accommodate for the increased variation in context.

In contrast to residents and attending physicians, analysis of medical student transcripts revealed two categories not present in studies of resident and board certified physicians: anchoring and misinterpretation of data. Anchoring manifested as an underestimation of the value of new data versus misinterpretation of data that manifested as utterances of not knowing how to value the data. Medical students demonstrated both tendencies. These two themes may relate to script theory [14]. Medical students would be expected to have less developed illness scripts than residents and practising internists. Expressions of diagnostic uncertainty and anchoring may be related to their predicted less robust script development.

The traditional view of contextual factors is that they have been seen as extrinsic to the clinical scenario. In previous work in board certified physicians [2], we tried to understand how contextual factors could play a distracting role by complicating a clinical case as a factor extrinsic to the clinical scenario. The impact of these contextual factors and findings in board certified physicians mirrored resident physicians. Through these studies [2, 6], the data suggests that a contextual factor can be innate to the clinical scenario, such as being able to diagnose colorectal cancer in a patient who does not speak English well, which is consistent with situated cognition. However, there may be contextual factors that remain extrinsic to the clinical scenario, such as a disrupting telephone call, which impact clinical reasoning by means of increasing cognitive load. As such, this would contend that expertise is on one hand a tolerance for extraneous cognitive load [9] and the ability to include contextual factors into the intrinsic nature of the clinical scenario in one’s own illness script.

Potential implications for education include the value of deliberate practice and illness script formation. This was seen through the changes in performance from students to residents in response to the contextual factors [6]. Our findings also provide some preliminary evidence to support the importance of medical knowledge in medical student education, which is consistent with script theory and the development of expertise. Further, as all groups were impacted by patient and system factors [2, 6], our findings suggest that additional attention and training in the development of strategies to mitigate the impact of contextual factors as extraneous cognitive load could improve learning and performance. This is consistent with situated cognition theory.

This study had several limitations. First, the medical students watched video recorded cases. This may not always allow the medical student to reframe the encounter easily and could impact how clinical reasoning processes occur. In addition, the watching of video recorded clinical encounters may not translate completely to what occurs in acute patient care settings. Evaluating the impact of contextual factors on clinical reasoning during an objective structured clinical exam may potentially help to mitigate both of these factors. Second, a small number of participants were sampled from one medical school, which may limit the generalizability of the findings. In addition, a sampling bias cannot be excluded given this small number, which is typical for clinical reasoning studies, and that the majority of participants were male. Third, as a novice, medical students may experience greater levels of anxiety during observed cognitive tasks, such as a research study, than a resident or board certified physician. As such, this may result in increased levels of uncertainty than what would be expected to occur in a non-observed setting.

There were several strengths to this study. First, consistent with data from resident physicians, there was universal acknowledgement of the contextual factors across all participants and all cases. This could suggest that contextual factors may be intrinsic to a clinical scenario. Second, the themes found demonstrated significant overlap with the data from other studies [2, 6], supporting previous findings in both resident and expert physicians. Third, despite a small number of participants, saturation was reached quickly and the coders demonstrated 100% agreement. Taken together, we believe this increases our insight into how contextual factors may impact clinical reasoning processes as viewed through the lens of situated cognition.

## Conclusion

We believe our work increases the understanding of how contextual factors impact clinical reasoning processes in medical students when viewed through the lens of situated cognition. This raises important questions about how a clinician processes contextual factors and whether the contextual factor represents an intrinsic versus extrinsic modifier to the clinical scenario or clinical reasoning process. Going forward, understanding how contextual factors impact cognitive load has implications for delineating how clinical reasoning processes can best be taught. In addition, understanding how to best account for contextual factors in the acquisition of illness scripts has implications for understanding how to best optimize clinical reasoning accuracy.

## References

[1] Higgs J, Jones M, Higgs J, Jones M, Loftus S, Christensen N (2008). Clinical decision making and multiple problem spaces. Clinical reasoning in the health professions.

[2] Durning S, Artino AR, Pangaro L, van der Vleuten CP, Schuwirth L (2011). Context and clinical reasoning: understanding the perspective of the expert’s voice. Med Educ.

[3] Norman GR, Tugwell P, Feightner JW, Muzzin LJ, Jacoby LL (1985). Knowledge and clinical problem-solving. Med Educ.

[4] Elstein AS, Shulman LS, Sprafka SA (1990). Medical problem: solving a ten-year retrospective. Eval Health Prof.

[5] Eva K, Neville A, Norman G (1998). Exploring the etiology of content specificity: factors influencing analogic transfer and problem solving. Acad Med.

[6] McBee E, Ratcliffe T, Picho K (2015). Consequences of contextual factors on clinical reasoning in resident physicians. Adv. Health. Sci. Educ. Theory. Pract..

[7] Durning SJ, Artino AR (2011). Situativity theory: a perspective on how participants and the environment can interact: AMEE Guide no. 52. Med Teach.

[8] Schunk D (2008). Learning theories: an educational perspective.

[9] Durning SJ, Artino A, Boulet J (2012). The feasibility, reliability, and validity of a post-encounter form for evaluating clinical reasoning. Med Teach.

[10] Durning S, Artino A, Van der Vleuten C, La Rochelle J, Arze B, Schuwirth L (2010). Making use of contrasting participant views of the same encounter. Med Educ.

[11] Ericsson K, Simon H (1980). Verbal reports as data. Psychol Rev.

[12] Kennedy T, Lingard L (2006). Making sense of grounded theory in medical education. Med Educ.

[13] Sweller J (1988). Cognitive load during problem solving: effects on learning. Cogn Sci.

[14] Feltovich PJ, Barrows HS, Schmidt HG, de Volder ML (1984). Isssues of generality in medical problem solving. Tutorials in problem-based learning: new directions in training for the health professions.

